# Small Cell Lung Cancer (SCLC) Presenting as Acute Pancreatitis: The Role of Hypercalcemia

**DOI:** 10.4021/wjon266w

**Published:** 2012-02-19

**Authors:** Jessica Tan, Alejandro Calvo

**Affiliations:** a Kettering Medical Center Internal Medicine Residency Program - Graduate Medical Education, Kettering, OH, USA; b Dayton Cancer Center -Kettering Health Network, Dayton, OH, USA

**Keywords:** SCLC, Hypercalcemia, Pancreatitis

## Abstract

A 53-year-old smoker female presented to our hospital with abdominal pain secondary to acute pancreatitis. Severe hypercalcemia was felt to be the precipitating cause of pancreatitis. Her work up showed SCLC with bone marrow metastases as the only site of extra-thoracic metastases. We review the literature regarding hypercalcemia mechanisms in patients with SCLC.

## Introduction

Hypercalcemia is a relatively common metabolic complication in patients with cancer, occurring in approximately 25 percent of cases. It occurs in patients with both solid tumors and hematologic malignancies. The most common cancers associated with hypercalcemia are breast, non-small cell lung cancer and multiple myeloma.

Hypercalcemia is rarely associated to SCLC even in the setting of bone metastases.

We review the case of a patient with newly diagnosed SCLC who presented with acute pancreatitis triggered by hypercalcemia of malignancy. Both events are very rare. The molecular mechanisms that mediate hypercalcemia in SCLC are not clear.

## Case Report

A 53-year-old Caucasian female was admitted for acute pancreatitis secondary to hypercalcemia. She reported constitutional symptoms of weight loss, altered taste and generalized weakness. Upon admission her calcium level was 15.5 mg/dl; phosphorus was 4.2 mg/dl.

Further laboratory work-up showed: Alkaline phosphatase 79; PTH was 4.4; PTHrP was < 1.1. TSH was normal, 1.25 (OH) vitamin D and 25-OH vitamin D were low. SPEP and UPEP were consistent with acute inflammation but no monoclonal proteins were present.

**Figure 1:**
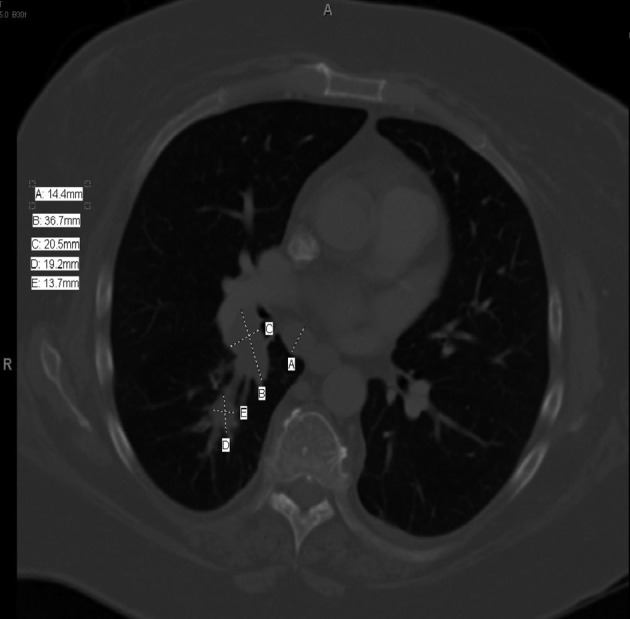
CT chest.

**Figure 2:**
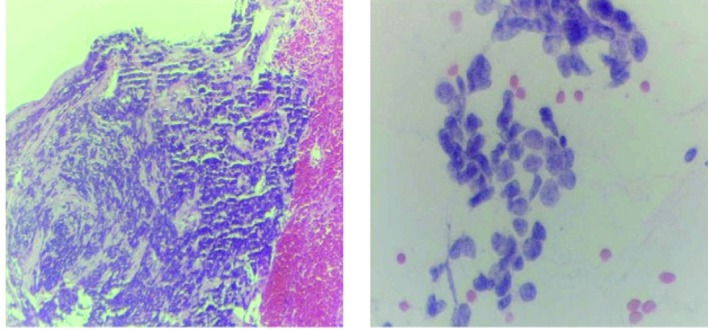
Bronchial brushing and biopsy.

CT Chest (Fig. 1) showed a 3.7 cm right hilar mass, bronchoscopic biopsy (Fig. 2) showed small cell lung carcinoma (SCLC). Staging work up ruled out brain, liver or adrenal lesions. Her bone scan was negative for metastases.

**Figure 3:**
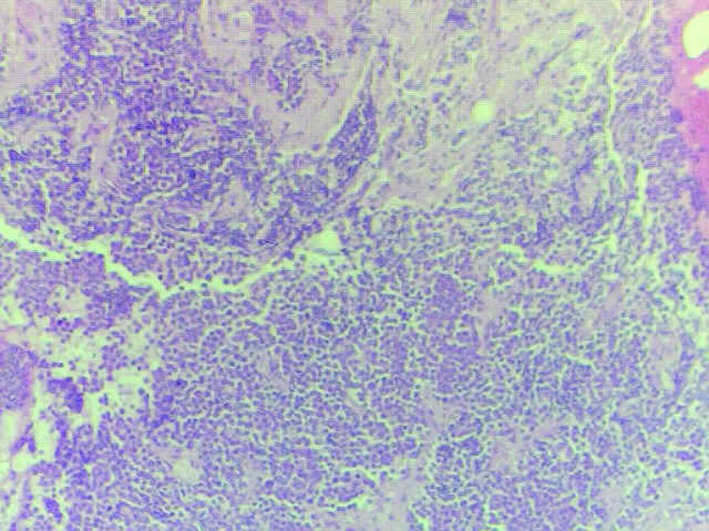
Bone marrow biopsy.

Due to significant anemia and thrombocytopenia, she had a bone marrow biopsy, which showed invasion with small cell carcinoma (Fig. 3).

The patient’s hypercalcemia did not respond to intravenous hydration alone. She required intravenous zoledronic acid to bring her calcium levels within normal range. She received her first round of platinum-based chemotherapy prior to discharge.

## Discussion

Hypercalcemia is a very unusual metabolic complication in SCLC patients. There are few reports in the literature that discuss hypercalcemia seen in this clinical scenario [1-8].

The mechanism of hypercalcemia in SCLC is unknown. There is an isolated case report of PTHrP-mediated hypercalcemia in a patient with SCLC [2]. In another case report, a patient had an elevated PTH associated to SCLC as the mechanism to explain hypercalcemia [1].

Based on our patient’s initial laboratory findings of hypercalcemia and normal phosphorus level, it was suspected that the hypercalcemia was not PTH-mediated. Her PTH level was indeed suppressed. PTHrP level was undetectable. Other causes of hypercalcemia were ruled out in our patient. She did not have excessive vitamin D levels, associated plasma cell dyscrasia, thyroid abnormalities or findings suggestive of granulomatous disease.

There is not a single unifying mechanism to explain the rare event of hypercalcemia in SCLC patients [3, 5]. The common denominator in most reports seems to be either bone or bone marrow involvement [4]. On the other hand we note that bone metastases are fairly common in SCLC but its association with hypercalcemia is not. Therefore we are left with the hypothesis that bone marrow invasion is the underlying mechanism to explain this unusual phenomenon. In our patient’s case we speculate that bone marrow invasion by SCLC was associated to cytokine production with subsequent osteoclastic stimulation. This in turn led to abnormal elevation in calcium levels. Possible mediators would be IL-6, RANK-L or TNF alpha. The management of hypercalcemia in these patients remains unchanged with IV fluids, loop diuretics and bisphosphonates.
